# De novo transcriptome datasets of *Shorea balangeran* leaves and basal stem in waterlogged and dry soil

**DOI:** 10.1016/j.dib.2019.104998

**Published:** 2019-12-14

**Authors:** Fitri Indriani, Ulfah J. Siregar, Deden D. Matra, Iskandar Z. Siregar

**Affiliations:** a Tropical Silviculture Study Program, Graduate School of IPB University (Bogor Agricultural University), Indonesia; b Department of Silviculture, Faculty of Forestry, IPB University (Bogor Agricultural University), Indonesia; c Department of Agronomy and Horticulture, Faculty of Agriculture, IPB University (Bogor Agricultural University), Indonesia

**Keywords:** *Shorea balangeran*, Transriptome, RNA-seq, Adaptation

## Abstract

*Shorea balangeran* Burk locally known as balangeran has been widely used as recommended species for tropical peat swamp forest restoration, due to the capability of these species to grow in waterlogged and dry areas. However, the information concerning genetic basis of adaptation to ecological condition variation is limited and no transcriptome study has been reported in this context. Here we reported two sets of transcriptome data from a sample of leaf and basal stem that were taken from seedlings growing in potted media containing peat and mineral soil. The raw reads are stored in the DDBJ platform with accession number DRA008633.

**Table:** Specifications Table

Subject	Agricultural and Biological Sciences: Forestry
Specific subject area	Molecular study in Forestry
Type of data	RNA Sequencing Data
How data were acquired	Illumina Hiseq 4000
Data format	Raw sequencing reads and assembled contigs
Parameters for data collection	Leaf and basal steam of balangeran seedlings planted in waterlogged peat, dry peat, waterlogged mineral soil and dry mineral soil
Description of data collection	Total RNA was sequenced using Illumina Hiseq 4000 platform in NovogenAIT, Singapore
Data source location	Bogor, West Java Indonesia
Data accessibility	Repository name: DDBJ (DNA Data Bank of Japan) Data identification number: DRA008633 Direct URL to data: https://ddbj.nig.ac.jp/DRASearch/submission?acc=%20DRA008633
Related research article	F. Indriani, D.D. Matra, U.J. Siregar, I.Z. Siregar Ecological aspects and genetic diversity of *Shorea balangeran* in two forest types of Muara Kendawangan Nature Reserve, West Kalimantan, Indonesia, Biodiversitas. 20 (2019) 482–488 https://doi.org/10.13057/biodiv/d200226

**Table:** 

**Value of the Data** • This is the first transcriptome data of *Shorea balangeran* from leaves and basal stem • This data is beneficial to elucidate the molecular mechanism and gene pathway of *Shorea balangeran* response to different ecological condition • This data allows further analysis to identify genes of interest that play roles in *Shorea balangeran* adaptation process

## 1 Data

*Shorea balangeran* (balangeran) belongs to Dipterocarpaceae family that is distributed in peat and heath forest in Indonesia [1]. In this study the *de novo* transcriptome assembly of balangeran is reported for the first time. The transcriptome data were obtained from leaves and basal stem of seedling that were growing in potted media containing each of peat and mineral soil. The high quality of mRNA extracted were sequenced using Illumina Hiseq 4000. The statistics of the reads and assembled sequences are presented in Table 1. The overview of transcriptome data were showed in Table 2. Analysis showed that 113,998 contigs (63.62%) had significant matches in nr NCBI database and 78,407 (43.49%) in Swiss-Prot database and 90,875 (50.40%) in TrEMBL database. Out of 180,291 merged contigs, a total 130,314 open reading frames (ORFs) were identified (Table 3) with 5prime partial ORFs type 31,209 (23.95%), 3prime partial 17,633 (13.53%) and complete ORFs type 64,374 (49.40%) were identified. In this study, microsatellite motifs from merged contigs were identified (Table 4), mononucleotides were the most abundant type (44,626, 70.30%), followed by trinucleotides (11,160, 17.58%) and dinucleotides (6,270, 9.88%).

**Table 1:** The properties of reads and assembled sequences of balangeran.

Features	Numbers		
	Leaf	Basal Stem	Merged^b^ (Leaf and Basal Stem)
Reads			
Number of reads	64,101,942	56,537,051	120,638,993
Number of bases	9,615,291,300	8,480,557,650	18,095,848,950
Number of post-trimming reads	62,400,243 (97.35)	54,917,915 (97.14)	117,318,158 (97.25)
Number of post-trimming bases	9,360,036,450 (97.35)	8,237,687,250 (97.14)	17,597,723,700 (97.25)
Transcripts^a^			
Number of transcript	279,598	574,875	–
Number of bases	175,610,736	342,696,076	–
Length range (bp)	201-16,510	201-16,960	–
Average (bp)	628.08	596.12	–
N50 (bp)	940	839	–
GC contents (%)	42.28	45.56	–
Contigs^b^			
Number of contig	187,297	440,665	180,291
Number of bases	118,677,247	252,486,917	197,305,352
Length range (bp)	201-16,510	201-16,960	201-17,014
Average (bp)	633.63	572.97	1094.37
N50 (bp)	918	762	1489
GC contents (%)	42.6	46.2	44.3

a Constructed by Trinity Program.

b Constructed by CAP3, cd-hit-est, and corset (only for merged contig) programs.

**Table 2:** Functional annotation of balangeran contigs using several database.

Database Source	Number (percentage)
Contig Number	180,291
Non-redundant protein (nr) NCBI	113,998 (63.62)
Non-redundant Nucleotide (nt) NCBI	53,407 (29.62)
Swiss-Prot UniProt	78,407 (43.49)
TrEMBL UniProt	90,875 (50.40)

**Table 3:** Open Reading Frames (ORFs) prediction characteristics of balangeran contigs using TransDecoder.

Features	Contigs Number (percentage)
ORF contig	130,314
ORFs Type :	
a. 5prime_partial	31,209 (23.95)
b. 3prime_partial	17,633 (13.53)
c. Internal	17,104 (13.13)
d. Complete	64,374 (49.40)

**Table 4:** Number and motif of microsatellite of balangeran contigs.

Motifs	Number of Contigs (percentage)		
	Leaf	Basal Stem	Merged
Mononucleotide	26,259 (72.93)	48,786 (68.83)	44,626 (70.30)
Dinucleotide	3939 (10.94)	6943 (9.80)	6270 (9.88)
Trinucleotide	5192 (14.42)	13,443 (18.97)	11,160 (17.58)
Tetranucleotide	421 (1.17)	1221 (1.72)	995 (1.57)
Pentanucleotide	142 (0.39)	292 (0.41)	267 (0.42)
Hexanucleotide	54 (0.15)	193 (0.27)	164 (0.26)

## 2 Experimental design, materials, and methods

Balangeran seedlings were treated and raised in the nursery of Department of Silviculture, Faculty of Forestry, IPB University Bogor for 6 months. Two seedlings were grown in peat soil in which each seedling planted in waterlogged peat and dry peat. Two seedlings were grown in mineral soil in which each seedling planted in waterlogged soil and dry soil. Total RNA was isolated from leaves and basal stem using Plant Total RNA mini kit (Geneaid) following the protocol. The quantity and integrity were evaluated using P360 Nanophotometer (Implen, München, Germany) and Bioanalyzer 2100 (Agilent Technologies). RNA samples had RNA integrity number (RIN) values between 7.4 and 8.6. The total RNA extracted were used for Illumina Hiseq 4000 sequencing following the protocol (NovogeneAIT, Singapore). The quality of raw data were examined using FastQC [2] then performed using Trimmomatic 0.39 to remove adaptor sequences, contamination and low-quality reads with default parameters (TruSeq3-PE.fa:2:30:10 SLIDINGWINDOW:4:5 LEADING:5 TRAILING:5 MINLEN:25) [3]. Pre-processed read from each leaves and basal stem were *de novo* assembled using Trinity v.2.3.2 with default parameter (minimum length = 200) [4], generated high quality contigs [5]. The each contigs from leaves and basal stem were reconstructed using CAP3 [6] and CD-HIT-EST v.4.6.8 [7]. The contigs form leaves and basal stem were merged and reconstructed using CAP3, CD-HIT-EST, and Corset program [8,9]. The database such as NCBI non-redundant (nr) (downloaded by October 1, 2018) and NCBI nucleotide sequence (nt) (downloaded by October 1, 2018), SwissProt and TrEMBL of UniProt (downloaded by September 14, 2018) were used to annotate the contigs using BLAST + program [10]. Open reading frames (ORFs) of contigs were predicted by the TransDecoder package (https://github.com/TransDecoder/TransDecoder), with the minimum ORF length of 100 bp [11]. Microsatellite discovery was analyzed using MISA software (http://pgrc.ipk-gatersleben.de/misa) with parameter (unit size-minimum repeats) as follows: 1–10, 2–6, 3–5, 4–5, 5–5, 6–5 and the interruptions (maximum difference between microsatellites) was 100 bases.
